# Supplementary material for: A multispectral 3D live organoid imaging platform to screen probes for fluorescence guided surgery
Source: EMBO Mol Med. 2024 Jun 3;16(7):3. doi: 10.1038/s44321-024-00084-4 (PMC11251264; doi:10.1038/s44321-024-00084-4)
Supplement: Supplementary file 1 — Table EV1 [file 44321_2024_84_MOESM1_ESM.docx]

**Table EV1 |** Mutational background and origin of PDO lines within the NB biobank.

| **Line** | **Tumour stage** | **Biopsy site** | **MYCN amplification** | **ALK mutation** | **Chromosomal alteration** |
| --- | --- | --- | --- | --- | --- |
| NBL129 | Relapse | Pleura | Yes (60%) | Yes | Partial loss 1p, partial gain 17q |
| NBL067 | Primary | Left adrenal gland | Yes (70%) | Yes | Partial loss 1p, partial gain 17q |
| NBL039 | Relapse | Subclavicular lymphoid right | Yes (87%) | Unknown | Partial loss 1p, 11q loss |
| AMC772 | Primary | Adrenal | No | No | 11q loss, 17q gain |
| AMC717 | Primary | Adrenal | Yes | No | 1p loss, 17q gain |
| 000IJY | Primary | Left adrenal gland | Yes | No | Partial loss 1p, 1p36 loss, partial gain 17q |
| 000GKX | Primary | Right adrenal gland | No | No | 17q gain |

MYCN, MYCN proto-oncogene BHLH transcription factor; ALK, anaplastic lymphoma kinase
