# Supplementary material for: A multispectral 3D live organoid imaging platform to screen probes for fluorescence guided surgery
Source: EMBO Mol Med. 2024 Jun 3;16(7):3. doi: 10.1038/s44321-024-00084-4 (PMC11251264; doi:10.1038/s44321-024-00084-4)
Supplement: Supplementary file 2 — Table EV2 [file 44321_2024_84_MOESM2_ESM.docx]

**Table EV2 |** Origin and (hormone) receptor status of PDO lines within the BC biobank.

| **Line** | **Original biobank ID** | **Tumour stage** | **Biopsy site** | **Diagnosis** | **ER receptor** (positive cells) | **PG receptor** (positive cells) | **HER2 over-expression** (IHC-score) |
| --- | --- | --- | --- | --- | --- | --- | --- |
| 10T | HUB-01-B2-001 | Primary | Left breast | Ductal carcinoma | 0% | 0% | +3 |
| 13T | HUB-01-B2-003 | Primary | Right breast | Ductal carcinoma | 0% | 0% | +1 |
| 27T | HUB-01-B2-011 | Primary | Right breast | Ductal carcinoma | 20% | 10% | 0 |
| 34T | HUB-01-B2-013 | Primary | Unknown | Ductal carcinoma | 0% | 0% | +1 |
| 36T | HUB-01-B2-060 | Primary | Right breast | Ductal carcinoma | 0% | 0% | 0 |
| 38T | HUB-01-B2-061 | Primary | Left breast | Adeno-  carcinoma | 0% | 0% | 0 |
| 62T | HUB-01-B2-031 | Primary | Right breast | Ductal carcinoma | 0% | 0% | +1 |
| 100T | HUB-01-B2-066 | Primary | Right breast | Ductal carcinoma | 0% | 0% | +3 |
| 169M | HUB-01-C2-152 | Metastasis | Left hip | N/A | 0% | 0% | 0 |

ER, Estrogen; PG, progesterone; HER2, human epidermal growth factor receptor 2; IHC, Immunohistochemistry; N/A, not applicable
