# Supplementary material for: A multispectral 3D live organoid imaging platform to screen probes for fluorescence guided surgery
Source: EMBO Mol Med. 2024 Jun 3;16(7):3. doi: 10.1038/s44321-024-00084-4 (PMC11251264; doi:10.1038/s44321-024-00084-4)
Supplement: Supplementary file 4 — Table EV4 [file 44321_2024_84_MOESM4_ESM.docx]

**Table EV4** **|** Details of antibodies and llama VHHs and conjugates used.

| **Target** | **Antibody** | **Clone** | **Supplier** | **Conjugation** | **Fluorophore** | **Stock**  **Conc.** | **Dilution/Dose** | **Application** | **Ref.** |
| --- | --- | --- | --- | --- | --- | --- | --- | --- | --- |
| **THY1** (CD90) | mouse anti-human CD90 | 5E10 | BD Biosciences | Directly | FITC | 0.50mg/ml | 1:200 | NB *in vitro* | (Fiegel *et al*, 2008; Sauzay *et al*, 2019) |
| **GD2** | Qarziba (Dinutuximab beta) |  | EUSA Pharma | Manual | Alexa Fluor™ 514 | 0.26mg/ml | 1:100 | NB, BC *in vitro* | (Sait & Modak, 2017; Yu *et al*, 2010; Wellens *et al*, 2020) |
|  |  |  |  |  | IRDye800CW |  | 1 nmol | NB *in vivo* |  |
| **L1CAM** (CD171) | mouse anti-human L1CAM | L1-OV198.5 | BioLegend | Directly | PE | 0.50mg/ml | 1:20 | NB *in vitro* | (Künkele *et al*, 2017; Inaguma *et al*, 2016) |
|  |  | 198.5 | Elthera | Manual | Alexa Fluor™ 555 | 0.80mg/ml | 1:50 | BC *in vitro* |  |
|  |  |  |  |  | IRDye800CW |  | 1 nmol | NB *in vivo* |  |
| **NCAM-1** (CD56) | llama anti-NCAM VHH | Q55c | QvQ | Manual | Alexa Fluor™ 594 | 2.78mg/ml | 1:300 | NB *in vitro* | (Winter *et al*, 2008; Wachowiak *et al*, 2008; Geller *et al*, 2020) |
|  |  |  |  |  | HiLyte 488 | 1.2mg/ml | 1:100 | BC *in vitro* |  |
| **ALCAM** (CD166) | mouse anti-human CD166 | 3A6 | Bio-rad | Directly | Alexa Fluor™ 647 | 1.00mg/ml | 1:50 | NB *in vitro* | (Wachowiak *et al*, 2016; Corrias *et al*, 2010) |
| **EPCAM**  (CD326) | mouse anti-human CD326 | 9C4 | BioLegend | Directly | Alexa Fluor® 594 | 0.50mg/ml | 1:100 | BC *in vitro* | (Boogerd *et al*, 2019) |
| **HER2**  (CD340) | llama anti-HER2 VHH | 1D5 | University Utrecht | Directly | Alexa Fluor™ 633 | 0.95mg/ml | 1:100 | BC *in vitro* | (Deken *et al*, 2020) |
| **EGFR** | llama anti-EGFR VHH | 7D12 | University Utrecht | Directly | Alexa Fluor™ 647 | 0.33mg/ml | 1:50 | BC *in vitro* | (Oliveira *et al*, 2012; Driel *et al*, 2014) |

**Note |** Antibodies that were obtained unconjugated were manually conjugated via either NHS-esters or maleimide.

**Table EV4 references**

Boogerd LSF, Boonstra MC, Prevoo HAJM, Handgraaf HJM, Kuppen PJK, Velde CJH van de, Fish A, Cordfunke RA, Valentijn ARPM, Scheltinga AGT van, *et al* (2019) Fluorescence-guided tumor detection with a novel anti-EpCAM targeted antibody fragment: Preclinical validation. *Surg Oncol* 28: 1–8

Corrias MV, Gambini C, Gregorio A, Croce M, Barisione G, Cossu C, Rossello A, Ferrini S & Fabbi M (2010) Different subcellular localization of ALCAM molecules in neuroblastoma: Association with relapse. *Anal Cell Pathol* 32: 77–86

Deken MM, Kijanka MM, Hernández IB, Slooter MD, Bruijn HS de, Diest PJ van, Henegouwen PMP van B en, Lowik CWGM, Robinson DJ, Vahrmeijer AL, *et al* (2020) Nanobody-targeted photodynamic therapy induces significant tumor regression of trastuzumab-resistant HER2-positive breast cancer, after a single treatment session. *J Control Release* 323: 269–281

Driel PBAA van, Vorst JR van der, Verbeek FPR, Oliveira S, Snoeks TJA, Keereweer S, Chan B, Boonstra MC, Frangioni JV, Henegouwen PMP van B en, *et al* (2014) Intraoperative fluorescence delineation of head and neck cancer with a fluorescent Anti‐epidermal growth factor receptor nanobody. *Int J Cancer* 134: 2663–2673

Fiegel HC, Kaifi JT, Quaas A, Varol E, Krickhahn A, Metzger R, Sauter G, Till H, Izbicki JR, Erttmann R, *et al* (2008) Lack of Thy1 (CD90) expression in neuroblastomas is correlated with impaired survival. *Pediatr Surg Int* 24: 101–105

Geller JI, Pressey JG, Smith MA, Kudgus RA, Cajaiba M, Reid JM, Hall D, Barkauskas DA, Voss SD, Cho SY, *et al* (2020) ADVL1522: A phase 2 study of lorvotuzumab mertansine (IMGN901) in children with relapsed or refractory wilms tumor, rhabdomyosarcoma, neuroblastoma, pleuropulmonary blastoma, malignant peripheral nerve sheath tumor, or synovial sarcoma—A Children’s Oncology Group study. *Cancer* 126: 5303–5310

Inaguma S, Wang Z, Lasota JP & Miettinen MM (2016) Expression of neural cell adhesion molecule L1 (CD171) in neuroectodermal and other tumors. An immunohistochemical study of 5155 tumors and critical evaluation of CD171 prognostic value in gastrointestinal stromal tumors. *Oncotarget* 7: 55276–55289

Künkele A, Taraseviciute A, Finn LS, Johnson AJ, Berger C, Finney O, Chang CA, Rolczynski LS, Brown C, Mgebroff S, *et al* (2017) Preclinical Assessment of CD171-Directed CAR T-cell Adoptive Therapy for Childhood Neuroblastoma: CE7 Epitope Target Safety and Product Manufacturing Feasibility. *Clin Cancer Res* 23: 466–477

Oliveira S, Dongen GAMS van, Walsum MS, Roovers RC, Stam JC, Mali W, Diest PJ van & Henegouwen PMP van B en (2012) Rapid Visualization of Human Tumor Xenografts through Optical Imaging with a Near-Infrared Fluorescent Anti–Epidermal Growth Factor Receptor Nanobody. *Mol Imaging* 11: 7290.2011.00025

Sait S & Modak S (2017) Anti-GD2 immunotherapy for neuroblastoma. *Expert Rev Anticancer Ther* 17: 889–904

Sauzay C, Voutetakis K, Chatziioannou A, Chevet E & Avril T (2019) CD90/Thy-1, a Cancer-Associated Cell Surface Signaling Molecule. *Front Cell Dev Biol* 7: 66

Wachowiak R, Mayer S, Kaifi J, Gebauer F, Izbicki JR, Lacher M, Bockhorn M & Tachezy M (2016) Prognostic Impact of Activated Leucocyte Cell Adhesion Molecule (ALCAM/CD166) in Infantile Neuroblastoma. *Anticancer Res* 36: 3991–5

Wachowiak R, Rawnaq T, Metzger R, Quaas A, Fiegel H, Kähler N, Rolle U, Izbicki JR, Kaifi J & Till H (2008) Universal expression of cell adhesion molecule NCAM in neuroblastoma in contrast to L1: implications for different roles in tumor biology of neuroblastoma? *Pediatr Surg Int* 24: 1361–1364

Wellens LM, Deken MM, Sier CFM, Johnson HR, Ortiz F de la J, Bhairosingh SS, Houvast RD, Kholosy WM, Baart VM, Pieters AMMJ, *et al* (2020) Anti-GD2-IRDye800CW as a targeted probe for fluorescence-guided surgery in neuroblastoma. *Sci Rep* 10: 17667

Winter C, Pawel B, Seiser E, Zhao H, Raabe E, Wang Q, Judkins AR, Attiyeh E & Maris JM (2008) Neural cell adhesion molecule (NCAM) isoform expression is associated with neuroblastoma differentiation status. *Pediatr Blood Cancer* 51: 10–16

Yu AL, Gilman AL, Ozkaynak MF, London WB, Kreissman SG, Chen HX, Smith M, Anderson B, Villablanca JG, Matthay KK, *et al* (2010) Anti-GD2 Antibody with GM-CSF, Interleukin-2, and Isotretinoin for Neuroblastoma. *N Engl J Med* 363: 1324–1334
