# Supplementary figures and images for: A multispectral 3D live organoid imaging platform to screen probes for fluorescence guided surgery
Source: EMBO Mol Med. 2024 Jun 3;16(7):3. doi: 10.1038/s44321-024-00084-4 (PMC11251264; doi:10.1038/s44321-024-00084-4)

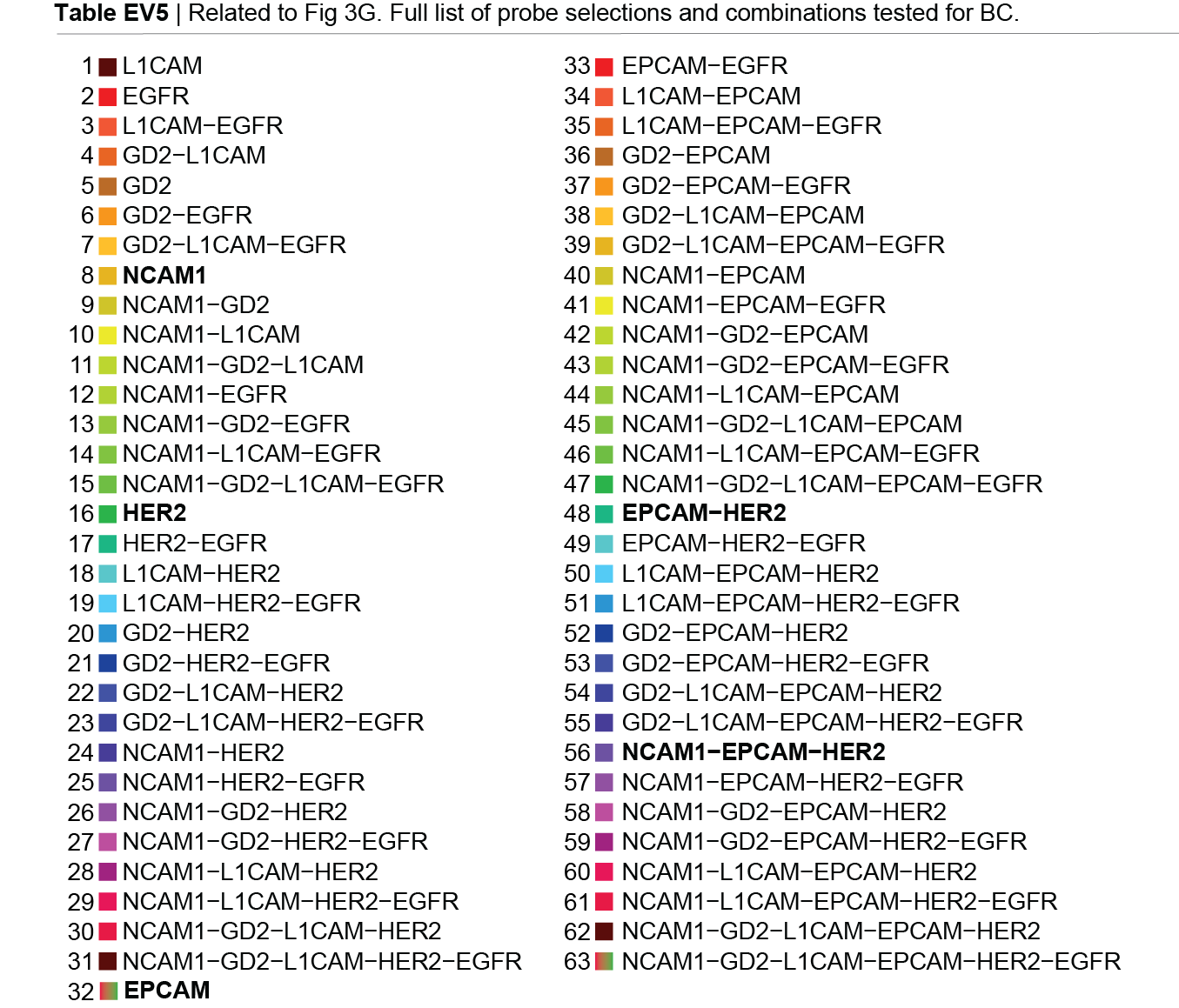

Supplement: Supplementary file 5 — Table EV5 [file 44321_2024_84_MOESM5_ESM.png]
