# Supplementary material for: A multispectral 3D live organoid imaging platform to screen probes for fluorescence guided surgery
Source: EMBO Mol Med. 2024 Jun 3;16(7):3. doi: 10.1038/s44321-024-00084-4 (PMC11251264; doi:10.1038/s44321-024-00084-4)
Supplement: Supplementary file 7 — Movie EV1 [file 44321_2024_84_MOESM7_ESM.zip › Jeremiasse_caption Movie EV1.docx]

Caption Movie EV1_Jeremiasse *et al*. (EMM-2023-18863-V3)

**Movie EV1: Movie summarizing the key features of the organoid-based multi-spectral 3D imaging platform and how it was applied for FGS probe identification.**
