# Supplementary material for: A multispectral 3D live organoid imaging platform to screen probes for fluorescence guided surgery
Source: EMBO Mol Med. 2024 Jun 3;16(7):3. doi: 10.1038/s44321-024-00084-4 (PMC11251264; doi:10.1038/s44321-024-00084-4)
Supplement: Supplementary file 10 — Expanded View Figures [file 44321_2024_84_MOESM10_ESM.pdf]

Expanded View Figures

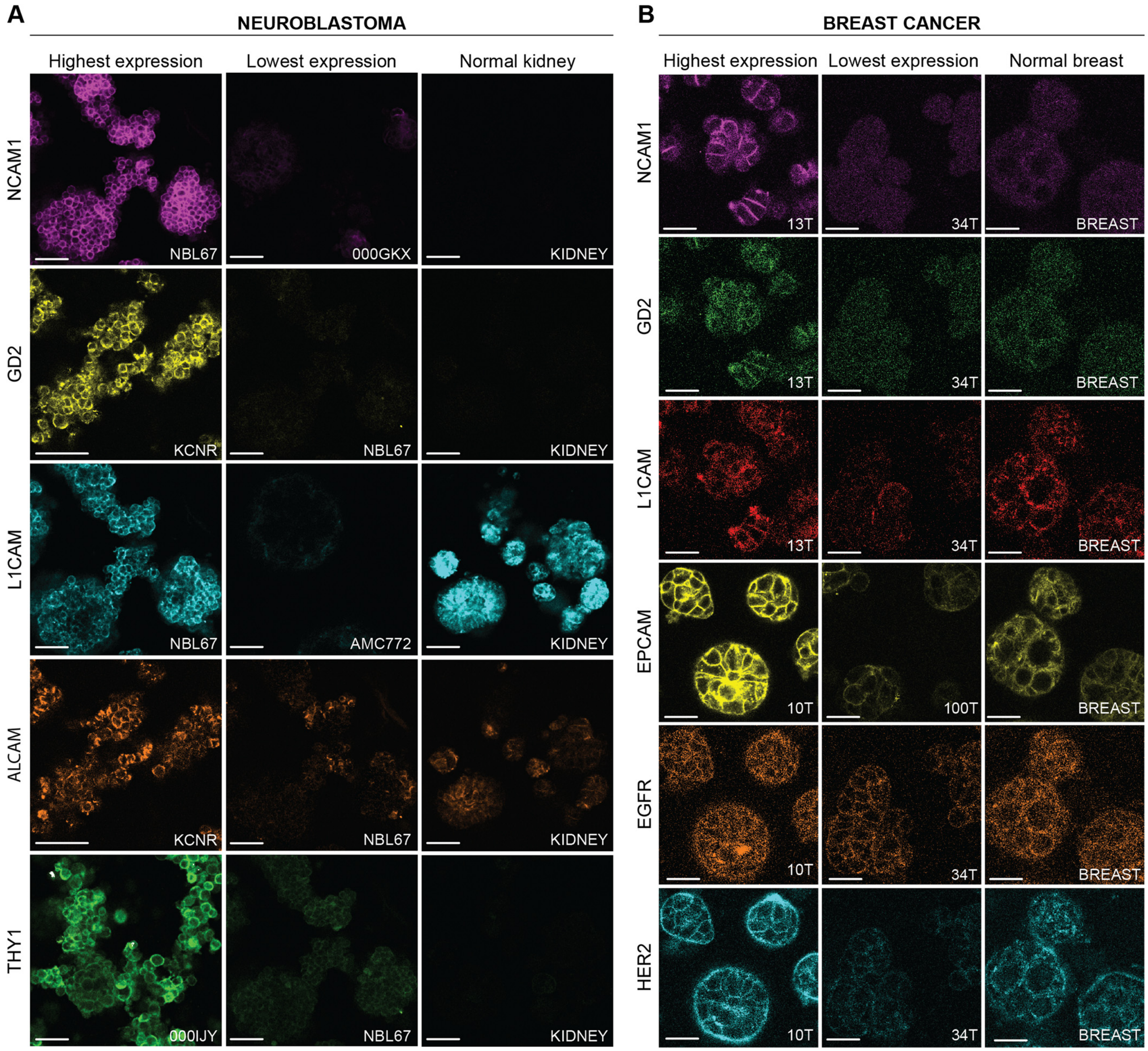

Figure EV1. Representative single-channel optical sections.

(**A**, **B**) Images of optical sections representing the highest (left) and lowest (middle) expression of the screened probes and expression on a healthy tissue control organoid line (right) for NB (**A**) and BC (**B**). Scale bars 50 μm (**A**) and 30 μm (**B**).

© The Author(s)

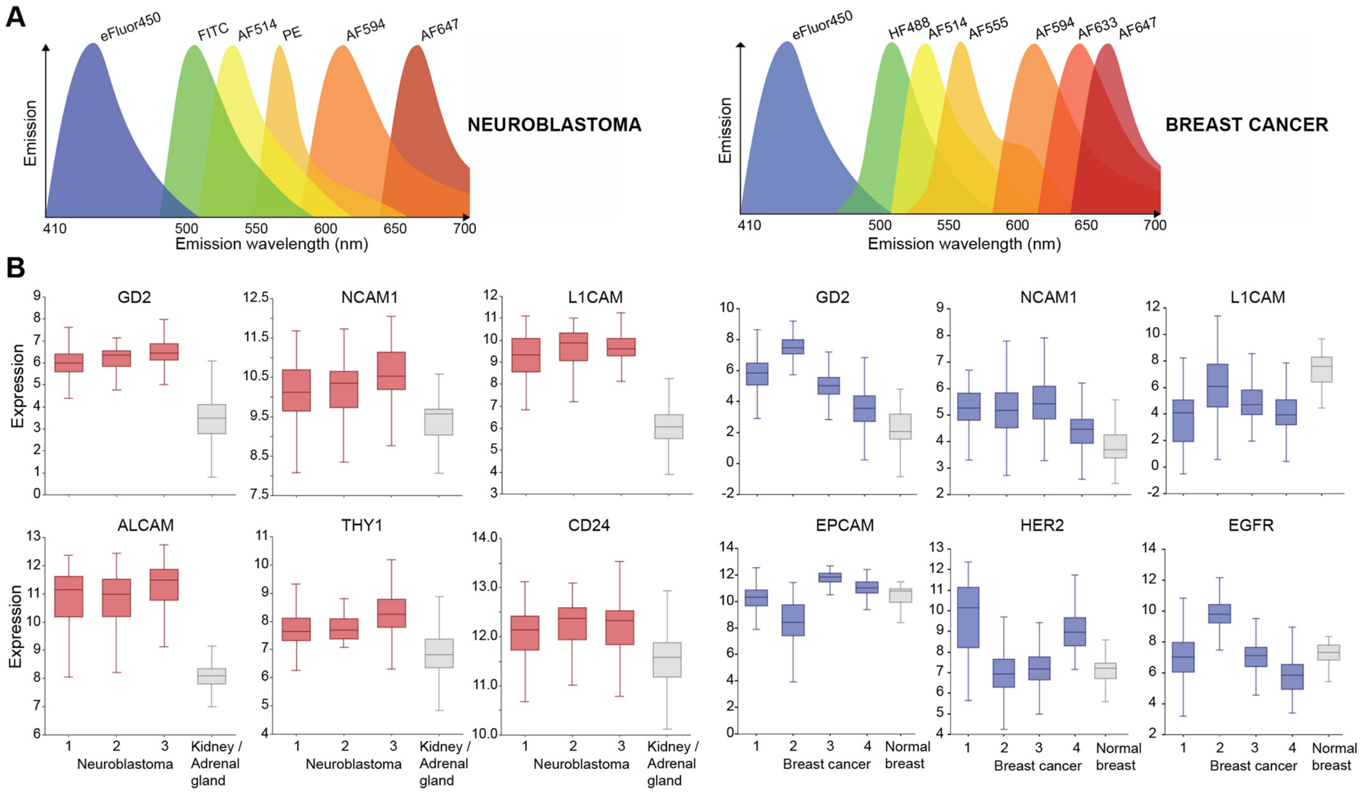

Figure EV2. Emisson spectra and target selection.

(**A**) Schematic representation of the fluorophore emission spectra for 7-color imaging of NB (left) and BC (right). (**B**) Box plot depicting relative target gene expression from R2 RNA sequencing datasets for NB (left) and BC (right) and respective adjacent healthy control tissue. Centre: median, bounds: Q1–Q3, whiskers extend to minimum/maximum limited to 1.5 times the IQR. Details of sequencing datasets, including sample size, is provided in Methods.

 © The Author(s)

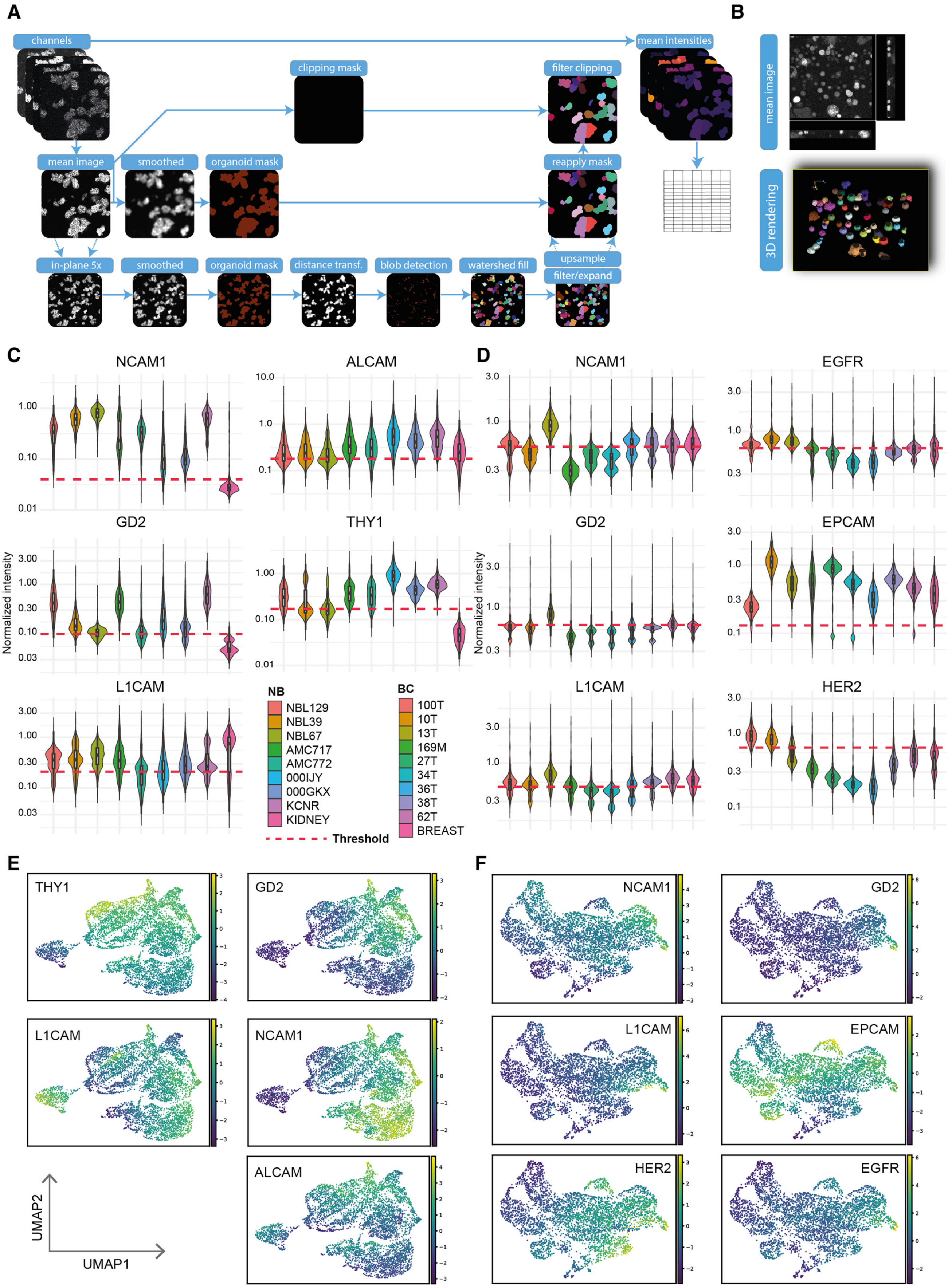
A
channels
mean intensities
clipping mask
filter clipping
mean image
smoothed
organoid mask
reapply mask
in-plane 5x
smoothed
organoid mask
distance transf.
blob detection
watershed fill
upsample
filter/expand
B
mean image
3D rendering
C
NCAM1
ALCAM
GD2
THY1
L1CAM
Normalized intensity
NB
NBL129
NBL39
NBL67
AMC717
AMC772
000IJY
000GKX
KCNR
KIDNEY
BC
100T
10T
13T
169M
27T
34T
36T
38T
62T
BREAST
Threshold
D
NCAM1
EGFR
GD2
EPCAM
L1CAM
HER2
Normalized intensity
E
THY1
GD2
L1CAM
NCAM1
ALCAM
UMAP2
UMAP1
F
NCAM1
GD2
L1CAM
EPCAM
HER2
EGFR

◀ **Figure EV3. STAPL-3D pipeline optimized for individual organoid segmentation and fluorescent intensity extraction.**

(**A**) Schematic representation of the STAPL-3D pipeline with key optimization steps for single organoid segmentation. (**B**) Representative raw 3D imaging data and associated 3D rendered single organoid segmentation. (**C**, **D**) Violin plots of the mean fluorescent intensities (MFI) of the screened probes for all individual organoids from the NB (**C**) and BC (**D**) PDO biobanks normalized for the experimental day and probe. Dashed line indicates the cut-off used for considering an organoid as positively stained. Boxplots inside violin plots; centre: median, bounds: Q1–Q3, whiskers extend to minimum/maximum limited to 1.5 times the IQR. $n = 3$ independent experiments. (**E**, **F**) Representation of the spatial target distribution used for UMAP clustering of both the NB (**E**) and BC (**F**) PDO biobank. $n = 3$ independent experiments.

 © The Author(s)

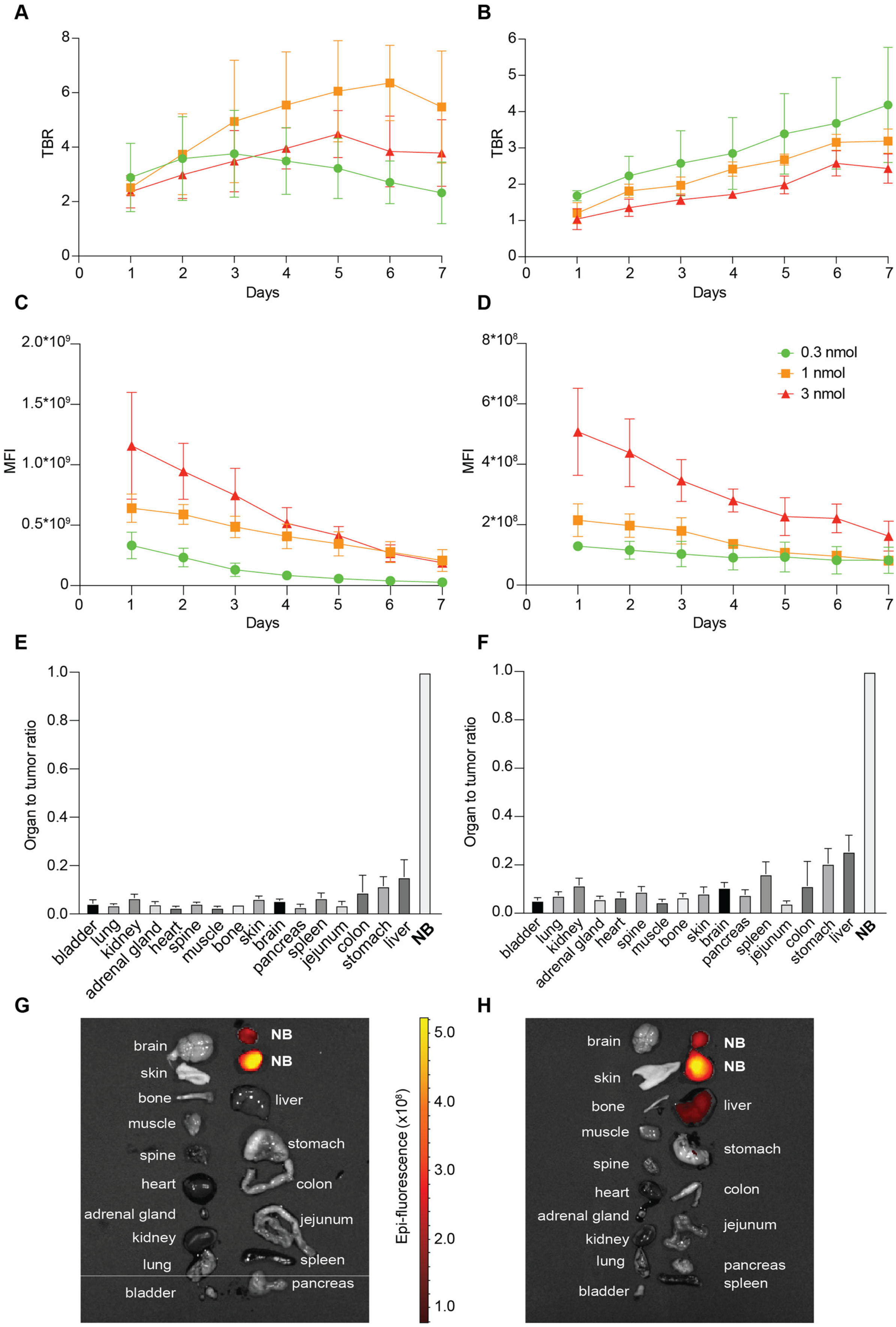
A
B
C
D
E
F
G
H
TBR
Days
MFI
0.3 nmol
1 nmol
3 nmol
Organ to tumor ratio
bladder
lung
kidney
adrenal gland
heart
spine
muscle
bone
skin
brain
pancreas
spleen
jejunum
colon
stomach
liver
NB
Epi-fluorescence (x10⁸)
5.0
4.0
3.0
2.0
1.0

Figure EV4. In vivo testing of anti-GD2-IRDye800CW and anti-L1CAM-IRDye800CW in NB xenografts.

(**A–D**) Line graph depicting the TBR (**A**, **B**) and MFI (**C**, **D**) of anti-GD2-IRDye800CW (**A**, **C**) and anti-L1CAM-IRDye800CW (**B**, **D**) per dose on 7 consecutive days. Mean TBR or MFI ± SD as imaged with the IVIS Spectrum system, $n = 3$ to 4 mice per dose group. (**E**, **F**) Bargraphs of the biodistribution of anti-GD2-IRDye800CW at day 5 (**E**) and anti-L1CAM-IRDye800CW at day 6 (**F**) in subcutaneous NB tumor-bearing mice receiving a 1 nmol dose. Mean MFI of organs and tissues normalized to the tumor + SD of $n = 3$ mice per probe. (**G**, **H**) Representative images of the biodistribution of anti-GD2-IRDye800CW at day 5 (**G**) and anti-L1CAM-IRDye800CW at day 6 (**H**) in subcutaneous NB tumor-bearing mice receiving a 1 nmol dose.

 © The Author(s)

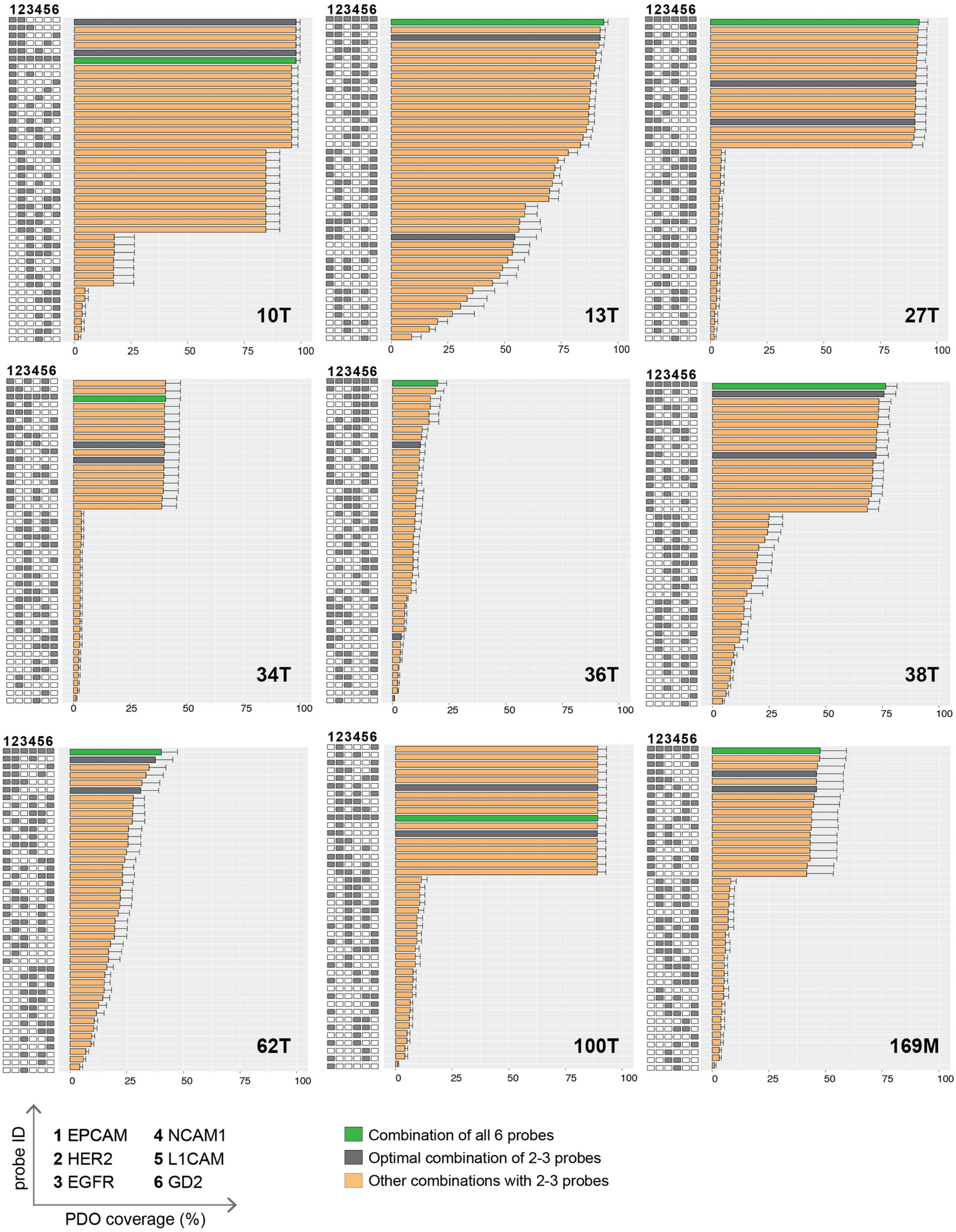
123456
10T
13T
27T
34T
36T
38T
62T
100T
169M
0
25
50
75
100
probe ID
PDO coverage (%)
1 EPCAM
2 HER2
3 EGFR
4 NCAM1
5 L1CAM
6 GD2
Combination of all 6 probes
Optimal combination of 2-3 probes
Other combinations with 2-3 probes

Figure EV5. Percentage organoid coverage with single probes and probe combinations per BC PDO line.

Percentage BC organoids that are positive for individual probes or probe combinations. Gray bars depict the 2 overall most effective probe combinations with a limited number of probes. In case of similar percentages, combinations are ranked higher if they consist of less probes. Green bars represent the maximum achievable percentage of coverage obtained with the combination of all six probes tested. Bars depict mean percentage + SEM of $n = 3$ independent experiments.

© The Author(s)
